# Muscular Rheumatism

**Published:** 1894-06

**Authors:** 


### Selections and Abstracts.

Muscular Rheumatism.—The following abstract in the *British Medical Journal* of an article by Leube is from the *Deutsche Med. Woch.*, in which he argues that muscular rheumatism is not a local disease, but a general infective disorder with special localizations in the muscular system. The mode of onset of the disorder varies, but is sometimes marked by shivering and prodromal fever, and by *malaise* of some duration. In some cases the muscular pains may be widespread, and occasionally endocarditis is observed. Taking a series of about 200 cases, he found that fever was present in about one-third; it was seldom higher than 102° F., and generally fell after two days in hospital, either rapidly or after some irregular fluctuations. In one-sixth of the cases there was a cardiac murmur at the time of admission; it is generally supposed that endocarditis, which is so common a "complication" of joint rheumatism, scarcely ever occurs in relation with muscular rheumatism. It is not possible to say in how many of the cases in which a murmur was observed this was present before the onset of the muscular rheumatism, but it was noticed that in half the cases the murmur grew fainter or disappeared while the patient was under treatment, and that whereas in all the cases together fever was observed in one-third only, it was present in two-thirds of those in which there was a murmur. Moreover, in a few cases the murmur was observed to appear after the onset of the muscular rheumatism. Three such cases are related by Leube, but in one there was at a later stage muscular rheumatism, and in another the patient, at the time the muscular rheumatism came on, was under treatment for gonorrheal urethritis, vaginitis, and cervical endometritis. Leube states that he has seen joint rheumatism come on after muscular rheumatism in several cases, and points out that muscular rheumatism is frequently observed after affections of the joints. Pleurisy was in a few rare cases observed as a complication of muscular rheumatism, but albuminuria only once. Leube concludes that it is highly probable that the infective material in muscular rheumatism is an attenuated form of the virus of acute rheumatic arthritis. In Würzburg, where his observations were made, the cases of muscular rheumatism coming to the clinic were, as a rule, few in number and isolated, but at one time recently a large number of cases applied, so that half a ward was filled with them; this would appear to indicate an epidemic influence. He only mentions the question of treatment incidentally, but would seem to have relied on salicylate of sodium.—*North American Practitioner.*

